# Attenuation of Collagen-Induced Arthritis in Rat by Nicotinic Alpha7 Receptor Partial Agonist GTS-21

**DOI:** 10.1155/2014/325875

**Published:** 2014-02-27

**Authors:** Yiping Hu, Ruoxi Liu, Jinchao Li, Ye Yue, Wenxiang Cheng, Peng Zhang

**Affiliations:** ^1^Center for Translational Medicine Research and Development, Shen Zhen Institute of Advanced Technology, Chinese Academy of Science, Shen Zhen, Guangdong 518055, China; ^2^Department of Orthopaedics, Shandong University of Traditional Chinese Medicine, Shangdong 250014, China

## Abstract

This research was performed to observe the effect of GTS-21 on Collagen Induced Arthritis (CIA). CIA model was used and after the onset of arthritis, the rats were divided into three groups based on their clinical symptoms score. Two groups were intraperitoneally (IP) injected daily with GTS-21 (1 mg/kg, 2.5 mg/kg) for a week, whereas phosphate buffered saline (PBS) was used for the control group. Cytokine titers, radiological, and histological examinations were performed at different time points after treatment with GTS-21. Compared with those of the control, the levels of TNF-**α**, IL-1, and IL-6 in the serum were significantly reduced after GTS-21 management. In addition, radiological results show that bone degradation was inhibited as well. Moreover, the hematoxylin and eosin (H&E) staining indicated that the histological score was significantly alleviated in the therapeutic group. Tartrate-resistant acid phosphatase (TRAP) stain-positive cells were also detected in the destruction of the articular cartilage, which was significantly reduced compared with the control group. This study provides the first evidence on the effect of GTS-21 as a potential treatment for RA.

## 1. Introduction

Rheumatoid arthritis (RA) is a systemic autoimmune disorder of unknown etiology that mainly targets diarthrodial joints, synovial membrane first and then cartilage, ligaments, and subchondral bone [1]. The impact of RA is very important in terms of articular pain [2], patients' functional disability [3], and survival [4, 5], in particular when considering the occurrence of extraarticular manifestations [6, 7]. It has been reported that pro- and anti-inflammatory cytokines derived predominantly from cells of the macrophage lineage have a major role in the initiation and perpetuation of the chronic inflammatory process in the RA synovial membrane [8]. Therefore, several treatment methods for RA aim to block certain cytokines noted to be essential in the development of RA [9–11]. However, none of these methods can cure this disease at the current stage.

In the last decades, the treatment of RA is deeply changed; now it is well established that early treatment is mandatory in order to improve patients' prognosis and reach disease remission, which is the main goal that clinicians should now achieve [12–14]. The availability of new effective drugs that are able to modulate the inflammatory cascade of RA is another factor that contributes to this “change of perspective” in RA treatment; these drugs are generally known as bio(techno)logical agents and may target TNF [15], CD20+ cells [16], IL-6 [17], and T-cells costimulation [18]. The literature about these drugs is steadily increasing, in terms not only of effectiveness and safety profile description [19, 20], but also of responsiveness prediction [21, 22]. Despite the progresses, the number of unsatisfied needs in RA treatment still remains high, as the large number of biological agents under development suggests [23]. Therefore, new therapeutic methods for RA treatment are of great importance.

The nervous system has been demonstrated to be an important regulator of the immune system, and neuronal anti-inflammatory mechanisms have been selected by evolution to modulate inflammatory responses [24, 25]. These mechanisms can provide a major advantage for novel pharmacological anti-inflammatory strategies that control systemic inflammation [26]. Recent research indicated that acetylcholine, the principal neurotransmitter of the vagus nerve, is a key mediator of this cholinergic anti-inflammatory pathway. The neuronal nicotinic acetylcholine receptors (nAChRs) are named based on their subunit components, in which nicotinic *α*-7 acetylcholine receptor (*α*7nAChR) is a subunit of nAChRs [27]. The *α*7nAChR has been considered important for immune regulation in the absence of nerves; however, little is known about its therapeutic role in chronic joint inflammation. Interestingly, overexpressed *α*7nAChR in synovial biopsies from patients with RA may be a target in RA therapy [28].

Based on the above mentioned selective pharmacological stimulation of *α*7nAChR, it may have therapeutic potential for the treatment of inflammatory conditions. Consequently, more specific agonists of this receptor have been identified or developed and used in various studies. To date, one of the most effective *α*7 selective partial agonists for modulating inflammatory responses is GTS-21, which has been proven effective in attenuating the immune response and improving the outcome in animal models of pancreatitis [29], endotoxemia, sepsis [30], acute lung injury, and ischemia reperfusion injury [31–33]. GTS-21 has also been proven effective as an immunomodulatory drug that attenuates pro-inflammatory cytokine levels and improves survival in sepsis models [34], decreases severity in pancreatitis, and attenuates endotoxin-induced tumor necrosis factor (TNF) in lung tissue [31, 35].

However, no research was reported to identify the therapeutic effect of GTS-21 on RA. This study hypothesizes that *α*7nAChR provides a link between the neurologic system and the inflammatory process in the inflamed joint and that treatment with specific activators (GTS-21) of this receptor would reduce joint inflammation. For this purpose, we used a strain of rats susceptible to CIA, a widely used experimental model of RA, as it shares many histological and immunological features with this disease, such as pannus formation, bone and cartilage destruction, and synovitis as well [36]. In detail, CIA animal model in rat via intraperitoneally (IP) injecting the highly selective *α*7nAChR agonist GTS-21 was performed and the result was observed and analysed.

## 2. Materials and Methods

### 2.1. Animals

A total of 35 male Wistar rats (8 to 10 weeks of age) were purchased from Vital River Laboratory Animal Technology Co. Ltd. (Beijing, China). The animals were housed under special pathogen-free conditions at the animal facility of the Shen Zhen Institute of Advanced Technology, Chinese Academy of Science. The Institutional Animal Care and Use Committee of the Shen Zhen Institute of Advanced Technology, Chinese Academy of Science approved all of the experiments.

### 2.2. Inducing CIA in Rats

Arthritis was induced in 30 rats by treatment with type II collagen (5 normal rats as control); CIA was induced using a modified method previously described by Trentham et al. [37]. In brief, bovine collagen-II ((CII) Chondrex, 2002, USA, dissolved in 0.05 M acetic acid) was emulsified with an equal volume of incomplete Freund's adjuvant ((IFA) Chondrex, 7002, USA). This CII-IFA emulsified liquid was administered as a 0.2 mL intradermal injection at the dorsum of each rat's tail, approximately 2 cm distal from the base. At 10 days after the first immunization, each rat received 0.1 mL of CII-IFA booster via intradermal injection on the tail's ventral side. For arthritis assessment, all rats were monitored three times a week by the same person blinded to the treatment group, and the incidence of arthritis and clinical score were evaluated.

The severity of arthritis was assessed using an established semiquantitative scoring system of 0–4, where 0 = normal, 1 = swelling in 1 joint, 2 = swelling in >1 joint, 3 = swelling in the entire paw, and 4 = deformity and/or ankylosis [38]. The cumulative score for all four paws of each rat (maximum possible score of 16) was used to represent the overall disease severity and progression.

### 2.3. In Vivo Administration of GTS-21

To explore the effects of GTS-21 on the CIA rat model, GTS-21 was dissolved in PBS. 15 days after the second immunization, 24 of 30 induced rats showed symptoms of RA and they were divided into three groups, with each group comprising eight rats. The severity of arthritis score in these three groups was the same (each group score: 12) and treatment by drug that day after they were grouped, in which the two groups were receiving a once daily (IP) injection of GTS-21 (1.0 mg/Kg and 2.5 mg/Kg) for a week, whereas the other group was treated by PBS using the same method for a week.

### 2.4. Enzyme-Linked Immunosorbent Assay (ELISA)

TNF-*α*, IL-1, and IL-6 levels in the serum were determined after treatment with GTS-21 (7 and 20 days after the treatment), using a commercially available ELISA kits, according to the recommendations of the manufacturer (Neobioscience Technology Co., Ltd.).

### 2.5. Radiological Analysis

1, 2, and 3 months after being treated with GTS-21, radiographic scoring criteria (28 kv, 12 s, USA Fixitron X-ray) were assessed according to the method reported by Lin et al. [39]: 0 is normal intact bony outlines and normal joint space; 1 is slight abnormality with one or two exterior metatarsal bones showing slight bone erosion; 2 is definite early abnormality with bone erosion in three to five exterior metatarsal bones; 3 is medium destructive abnormality of all exterior metatarsal bones, as well as one to two interior metatarsal bones showing definite bone erosions; 4 is severe destructive abnormality of all the metatarsal bones showing definite bone erosion and at least one of the inner metatarsal joints completely eroded, leaving bony joint outlines partly preserved; and 5 is mutilated abnormality with the absence of decipherable bony outlines. All parameters were scored by at least two observers in a blind test manner.

### 2.6. Histological Analysis

3 months after the treatment, the rats were sacrificed and hind paws were fixed in 4.0% formalin for 12 hours and then decalcified in 10% EDTA (Sigma) for 20 days at room temperature. The serial paraffin sections (5 *μ*m) of the hind paws were stained with hematoxylin and eosin (HE) for assessment of synovial inflammation and bone erosions, with a leukocyte acid phosphatase staining kit (Sigma) for tartrate-resistant acid phosphatase (TRAP) to detect osteoclasts. All detailed processes were performed according to the recommendations of the manufacturer (Nanjing Jancheng Technology co., Ltd.).

To compare the histological differences among different foot joints, HE sections of different joints were evaluated using the following scale [40]: 0 is normal synovium; 1 is synovial membrane hypertrophy and cell infiltrates; 2 is pannus and cartilage erosions; 3 is major erosions of cartilage and subchondral bone; and 4 is loss of joint integrity and ankylosis. The assessment was performed by two independent investigators who were blinded to the identity of the specimens, and the average of the two scores was obtained. The sample size of each group was 16. TRAP-positive cells found in sections of the knee joints were collected. Specifically, collection of TRAP-positive cells found in six different microscopic fields per section were collected. Osteoclasts were quantified according to the following scores: 0 = normal (no osteoclasts), 1 = presence of a few osteoclasts (lining fewer than 5% of most affected bone surfaces), 2 = some osteoclasts (lining 5–25% of most affected bone surfaces), 3 = many osteoclasts (lining 30–50% of most affected bone surfaces), and 4 = abundant osteoclasts (lining >50% of most affected bone surfaces) [41].

### 2.7. Statistical Analysis

To evaluate the effects of different treatments, we determined the change in clinical arthritis scores in each mouse from the start of the treatment until the end of the experiment. A nonparametric test (Kruskal-Wallis test) was used to analyze the score data, including radiological and histological scores. The statistical significance level was set at a *P* value of 0.05 and 0.01. SPSS 17.0 was used for all experiments.

## 3. Results

### 3.1. CIA Model

**Figure 1:**
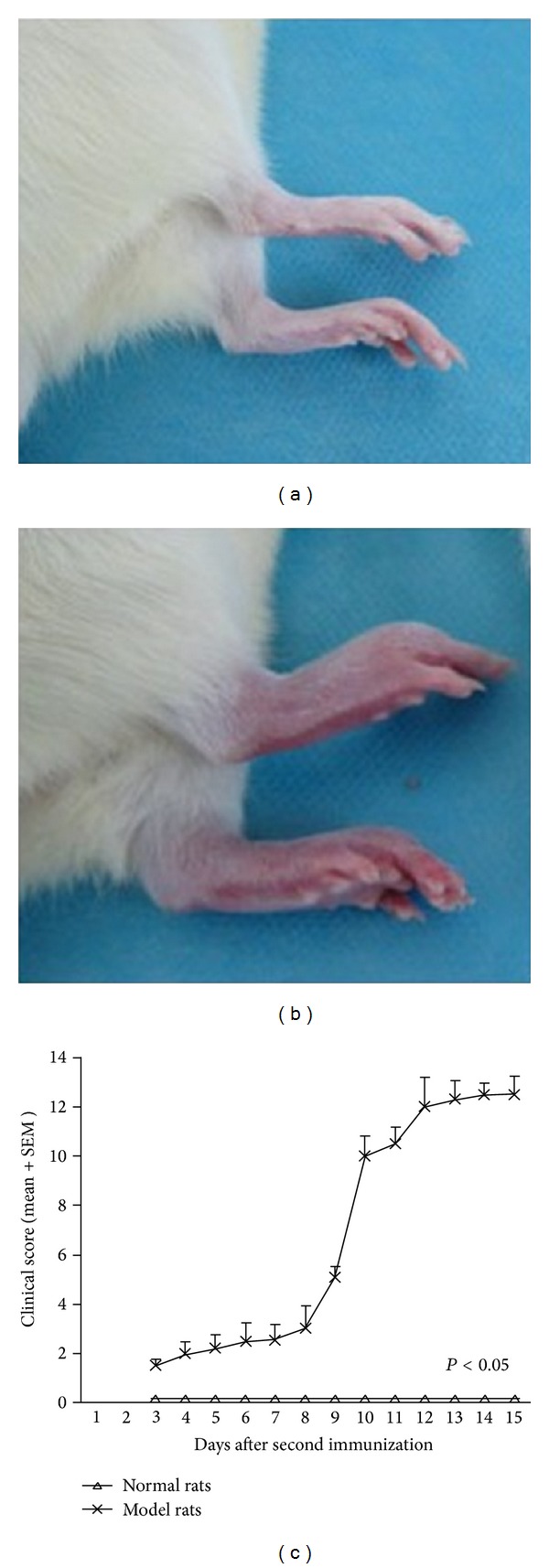
Macroscopic observation of joint swelling in rats with CIA. Arthritis was induced in 30 rats by treatment with type II collagen (5 normal rats as control), and the disease was scored clinically 3 times per week by the same person. (a) Normal rats, (b) arthritis onset of CIA model rats, and (c) the severity of arthritis clinical scoring. Differences between the control group and the model group were statistically significant (*P* < 0.05).

The macroscopic observation of joint swelling is shown in Figure 1. Joint swelling in the CIA model rats (Figure 1(b)) was significantly higher than that of the normal rats (Figure 1(a)). The severity of arthritis was assessed using an established semiquantitative scoring system, and the results are shown in Figure 1(c).

### 3.2. Cytokine Level in Peripheral Blood Serum

**Figure 2:**
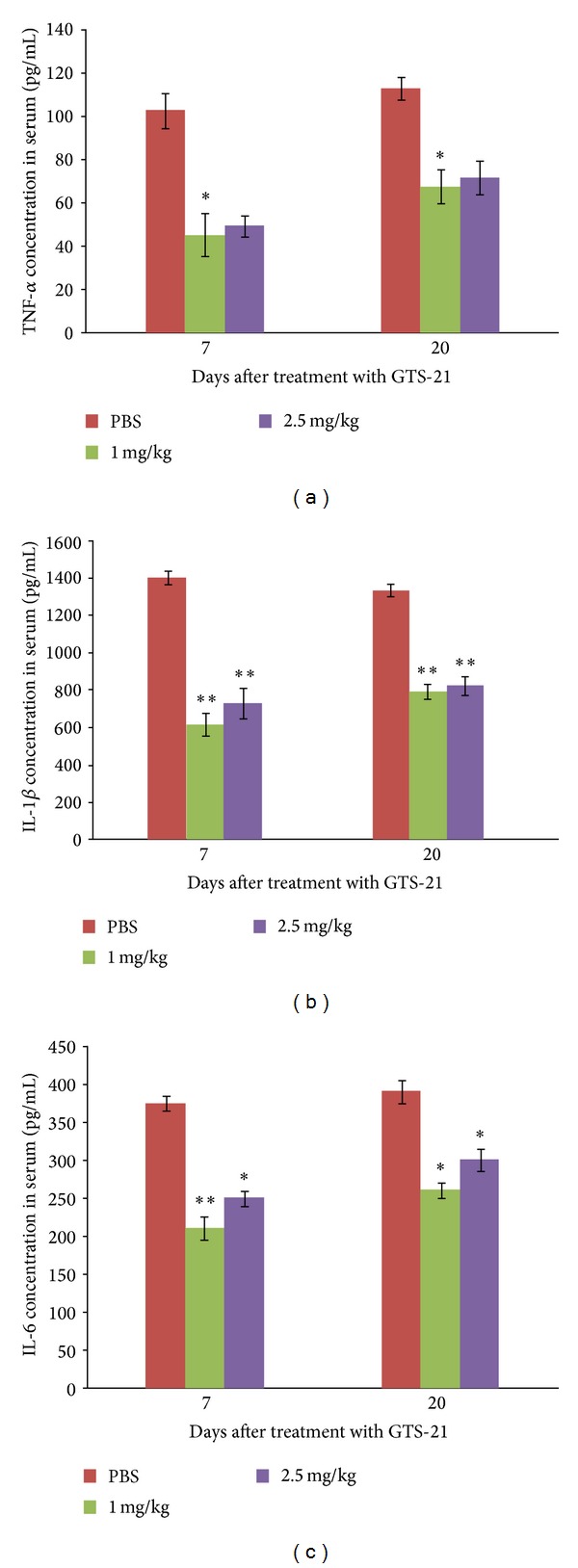
ELISA observation of inflammation-related cytokines. Serum concentrations of TNF-*α*, IL-1, and IL-6 are tested 7 and 20 days after treatment with GTS-21. (a) TNF-*α*, (b) IL-1, and (c) IL-6. *P < 0.05  **P < 0.01 versus treatment by PBS group.

After treatment with GTS-21 and PBS (7 and 20 days), the serum concentrations of TNF-*α*, IL-1, and IL-6 were tested using ELISA. The results showed that the cytokine levels in the treatment groups were significantly lower than those in the control group, as shown in Figures 2(a), 2(b), and 2(c).

### 3.3. Radiological Observation

**Figure 3:**
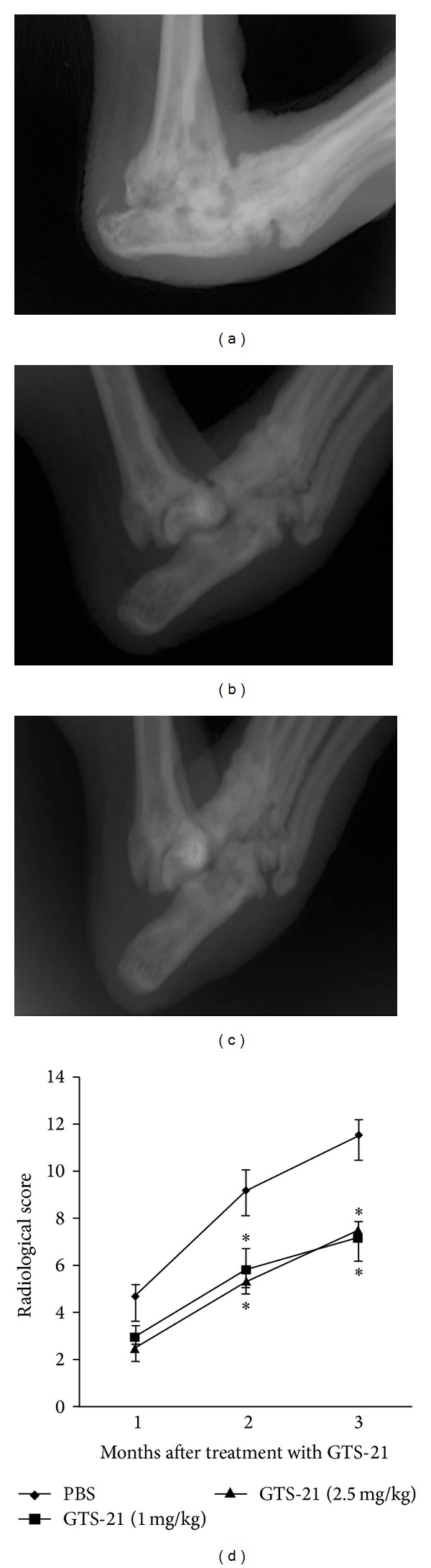
Inhibition of bone degradation in the joint by GTS-21 treatment. (a) Control group treated with PBS; (b) group treated with GTS-21 concentration of 1 mg/kg; (c) group treated with GTS-21 concentration of 2.5 mg/kg; (d) semiquantitative scoring of joint destruction. *P < 0.05 versus PBS control group. Data represent mean ± standard errors of the mean and are representative of eight rats per group.

Radiographs of the knee and foot joints were evaluated to investigate the effects of GTS-21 on bone degradation. The X-ray results were shown in Figures 3(a), 3(b), and 3(c). In the third month after treatment, in the PBS treated group normal joints in the knees and toes could barely be seen, but GTS-21 treated group significantly improved on joint destruction, whereas the semiquantitative scoring of joint destruction was shown in Figure 3(d). All the dates indicated that joint destruction was significantly reduced in the group treated with GTS-21.

### 3.4. Histological Analysis

**Figure 4:**
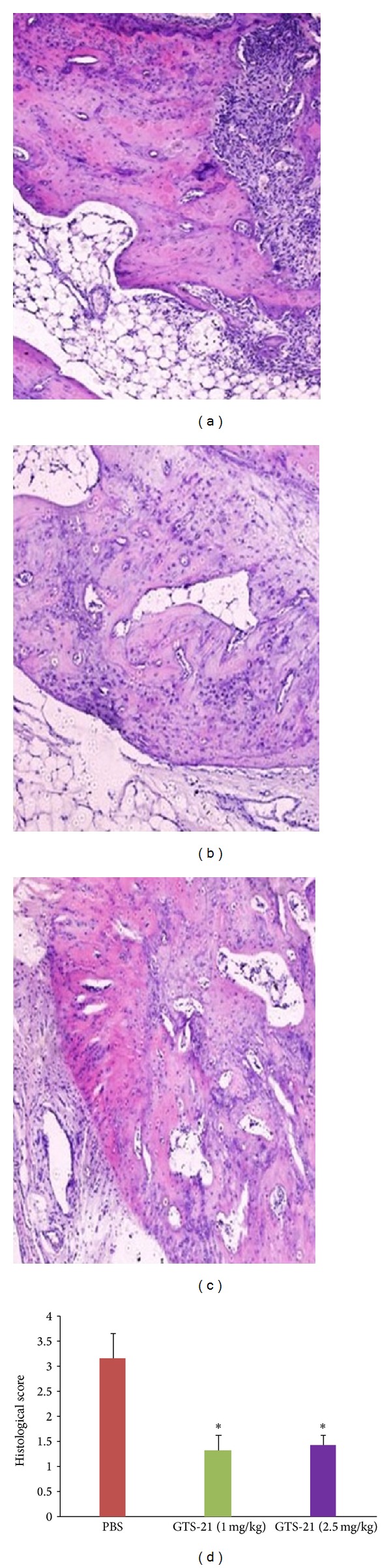
Histological observation of joint inflammation cell and bone damage. Tissue sections were stained with HE to study inflammatory cell influx and bone destruction (original magnification ×100). (a) Control group treated with PBS; (b) group treated with GTS-21 concentration of 1 mg/kg; (c) the group treated by GTS-21 concentration of 2.5 mg/kg; (d) histological score analysis of these three groups. Date was determined according to the scale described in Section 2.  *P < 0.05 versus PBS control group. Data represent mean + standard errors of the mean and are representative of eight rats per group.

**Figure 5:**
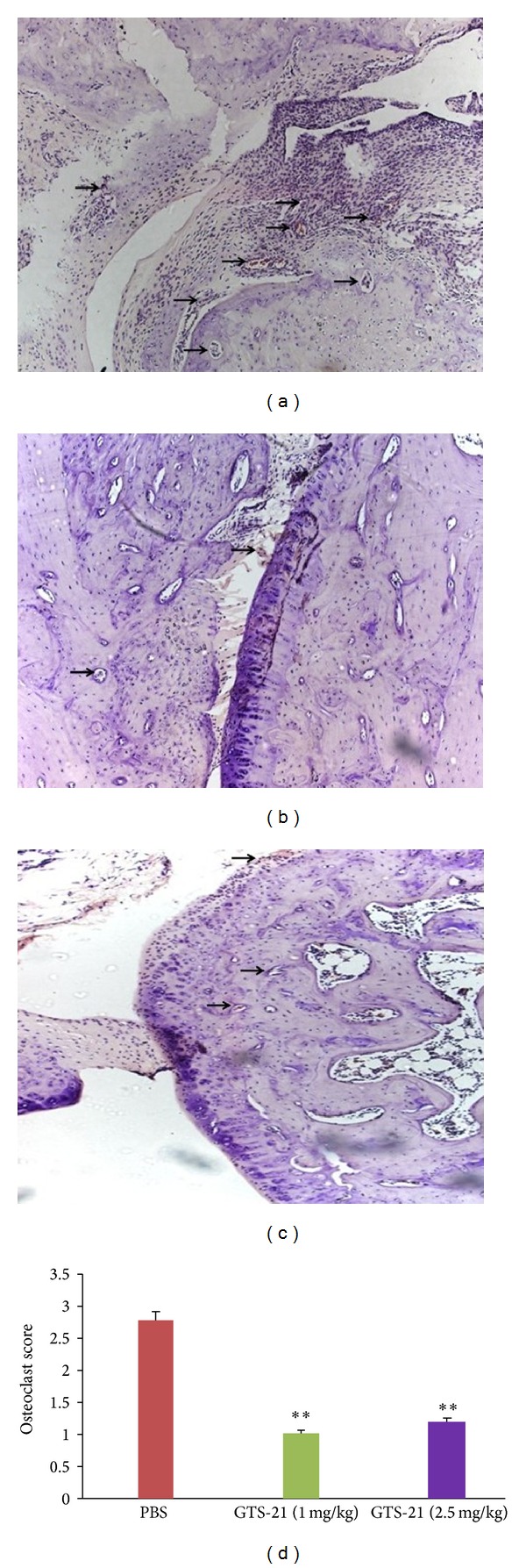
Osteoclast observation (the blank arrows). Tissue sections were stained with TRAP and restained with hematoxylin (original magnification ×100). (a) Control group treated with PBS; (b) group treated with GTS-21 concentration of 1 mg/kg; (c) group treated with GTS-21 concentration of 2.5 mg/kg; (d) statistical data of osteoclasts score in the knee joints of three different groups. **P < 0.01 versus PBS control group. Data represent mean + standard errors of the mean and are representative of eight rats per group.

Synovial inflammation and joint erosions were assessed by HE staining of ankle joint specimens, as shown in Figures 4(a), 4(b), and 4(c). Histologic scoring revealed a significant reduction of inflammatory cell infiltration in rats treated with GTS-21 compared with the control group (Figure 4(d)). In the knee joint, many TRAP stain-positive cells adhered to the eroded surface of the cartilage, which directly contributed to the erosion of such cartilage (Figures 5(a), 5(b), and 5(c)). Meanwhile, subchondral side erosion severity and pannus abundant were observed in the PBS treatment group. Moreover, it destroyed the joint from outside the cartilage. The score of osteoclasts in the knee joint of the treatment groups was significantly lower than that of the control group (Figure 5(d)). By contrast, no difference was observed between two different drug concentrations in the treatment groups with regard to histological and radiological scoring.

## 4. Discussion

Studies have indicated that *α*7nAChR is important for immune regulation [42]. Specific stimulation of *α*7nAChR on monocytes leads to efficient suppression of pro-inflammatory cytokine production. This receptor is essential for the efficient cytokine regulation in neuroimmune mechanisms known as the cholinergic anti-inflammatory pathway [43, 44]. RA is a chronic, inflammatory autoimmune disease of unknown cause and may be related to several signaling pathways. M. Westman et al. [28] reported the strong expression of *α*7nAChR in synovium of RA patients. These results indicated the importance of *α*7nAChR and cholinergic mechanisms in arthritis pathogenesis and implicated specific cholinergic modulation as a potential anti-inflammatory therapeutic strategy in joint inflammation.

GTS-21 is a derivative of the natural product anabaseine which is an effective portion of *α*7nAChR agonists. Moreover, GTS-21 is a characteristic *α*7nAChR-agonist that has been used in clinical trials and has been proven to be less toxic than nicotine [45, 46]. It has been reported that GTS-21 has been used in clinical trials to target neuronal *α*7nAChR in the brain of patients with Alzheimer's disease [47], since the cholinergic anti-inflammatory pathway is activated by stimulating the *α*7nAChR [33]. Meanwhile, the high expression of *α*7nAChR in the synovium of RA patients is a potential target of RA treatment and offers the possibility of GTS-21 as RA therapeutic drug.

In the present study, based on the newly discovered cholinergic anti-inflammatory pathway, we proposed a new method for the treatment of CIA using GTS-21. In our study, 7 and 20 days after treatment with GTS-21, those cytokines were significantly reduced relative to the control group. Based on observation of the inflammatory cell in the HE stained sections, the inflammatory environment was significantly improved in the treatment group. Furthermore, the treatment group had arthritis-related inflammatory cytokines that were much lower than those of the control group, which displayed significant inhibitory effects.

Bone and cartilage destruction are among the main symptoms of RA. We observed the destruction of knee joint at different times by radiology and measurement scores. As the disease developed, inhibitory effects were observed in the treatment group, opposite that of the control group, in which the joint was destroyed more seriously. This phenomenon was also observed in foot joints (results not shown). Osteoclast related erosion is one of the main factors of bone destruction in arthritis. In our observation of the osteoclast score, we found that the score of osteoclasts in the joints of the treatment group was lower than that of the control group. Many inflammatory cytokines, such as TNF-*α*, have already been proven to have important roles in osteoclast [48, 49]. Hence, the reduction of the secretion of cytokines in the treatment group could reduce the number of osteoclast, thereby reducing bone damage. Accordingly, we observed consistent results in our study, in which joint destruction was reduced in the treatments groups compared with the control group.

In summary, the results of the present study showed the anti-inflammatory effects of GTS-21 as a partial agonist of *α*7nAChR. GTS-21 may have a new function on the treatment of RA. However, our results showed that drug concentration has no obvious correlation with the effect of treatment. This observation may be attributed to the fact that the difference between the two concentrations of this study was not large enough. Using these questions, more in-depth study is needed in our next work. As the therapeutic value of GTS-21 is selective, this agonist may be a suitable candidate for development as a novel approach to RA treatment.
